# Effectiveness and safety of GLP-1 receptor agonists versus SGLT-2 inhibitors in type 2 diabetes: an Italian cohort study

**DOI:** 10.1186/s12933-022-01572-y

**Published:** 2022-08-24

**Authors:** Marta Baviera, Andreana Foresta, Pierluca Colacioppo, Giulia Macaluso, Maria Carla Roncaglioni, Mauro Tettamanti, Ida Fortino, Stefano Genovese, Irene Caruso, Francesco Giorgino

**Affiliations:** 1 Laboratory of Cardiovascular Prevention, Department of Health Policy, Istituto di Ricerche Farmacologiche Mario Negri IRCCS, Via Mario Negri 2, 20156 Milan, Italy; 2 Laboratory of Geriatric Epidemiology, Istituto di Ricerche Farmacologiche Mario Negri IRCCS, Milan, Italy; 3 Unità Organizzativa Osservatorio Epidemiologico Regionale, Lombardy Region, Milan, Italy; 4 Centro Cardiologico Monzino IRCCS, Milan, Italy; 5 Department of Emergency and Organ Transplantation, Section of Internal Medicine, Endocrinology, Andrology and Metabolic Diseases, University of Bari Aldo Moro, Bari, Italy

**Keywords:** Glucagon-like peptide-1 receptor agonists, Sodium-glucose cotransporter-2 inhibitors, Cardiovascular outcomes, Renal disease, Death

## Abstract

**Background:**

GLP-1 receptor agonists (GLP-1 RA) and SGLT-2 inhibitors (SGLT-2i) have shown to reduce the risk of major adverse cardiovascular events (MACE), death and worsening nephropathy when added to standard of care. However, these two dug classes differ in efficacy and safety. We compared the effectiveness and safety profile of GLP-1 RA and SGLT-2i in a large and unselected cohort of patients with type 2 diabetes resident in Lombardy from 2015 to 2020.

**Methods:**

Using linkable administrative health databases, we included patients aged 50 years and older initiating GLP-1 RA or SGLT-2i. Clinical events were: death, hospital admission for myocardial infarction (MI), stroke, heart failure (HF), and renal disease as individual and composite outcomes (MACE-3: all cause-death, non-fatal MI, non-fatal stroke; MACE-4: MACE-3 plus unstable angina). Outcomes were evaluated separately in subjects with and without previous cardiovascular (CV) diseases. Treatments were compared using Cox proportional hazards regression model after Propensity Score Matching (PSM) in both intention-to-treat (ITT) and per protocol (PP) analyses. Serious adverse events were also evaluated.

**Results:**

The analysis comprised 20,762 patients per cohort. The ITT analysis showed a significant risk reduction for non-fatal MI (HR 0.77; CI 95% 0.66–0.90), MACE-3 (HR 0.91; CI 95% 0.84–0.98), and MACE-4 (HR 0.92; CI 95% 0.86–0.99) in GLP-1RA compared with SGLT-2i users, while no difference was reported in the incidence of HF hospitalization and stroke between the two cohorts. Similar benefits were found in the subgroup of patients without previous CV diseases only. PP analysis largely confirmed the main results. The incidence of serious adverse events was low in both cohorts (< 1%).

**Conclusions:**

GLP-1RA showed to be equally safe and more effective than SGLT-2i in reducing the risk of MACE-3, MACE-4 and MI. This study adds to the growing body of real-world evidence addressing the specific clinical properties of GLP-1RA and SGLT-2i in everyday practice to tailor treatment to the individual patient.

**Supplementary Information:**

The online version contains supplementary material available at 10.1186/s12933-022-01572-y.

## Introduction

Cardiovascular outcome trials (CVOT) conducted over the past few years in patients with type 2 diabetes (T2D) have shown that GLP-1 receptor agonists (GLP-1 RA) and SGLT-2 inhibitors (SGLT-2i) reduced the risk of major adverse cardiovascular events (MACE), all-cause mortality and worsening nephropathy [1, 2]. However, GLP-1RA and SGLT-2i displayed several differences in terms of efficacy and safety. Specifically, in these placebo-controlled trials, GLP-1 RA were associated with a remarkable stroke risk reduction as opposed to the neutral effect of SGLT-2i, whereas SGLT-2i exerted a more prominent benefit on hospitalization for heart failure and adverse kidney outcomes [3–5]. With respect to safety and tolerability, gastrointestinal adverse events represent the most frequently reported side effects of GLP-1 RA [5], while genital infections, volume depletion and increased risk of ketoacidosis occur more frequently with SGLT-2i, sometimes leading to drug discontinuation [5, 6]. The subsequent evidence from real-world studies is in line with the results from CVOT, confirming the efficacy and safety profile of GLP-1 RA and SGLT-2i [7–10].

Recent guidelines recommend the initiation of GLP-1 RA or SGLT-2i in patients with high CV risk or in those with established atherosclerotic CV disease, regardless of baseline HbA1c levels [11]. Instead, SGLT-2i should be preferred in patients with heart failure or CKD and albuminuria [11]. Currently, there are no available head-to-head randomized trials comparing the effects of GLP-1RA and SGLT-2i on CV or renal outcomes; hence, despite their recognized limitations—such as the lack of randomization, low quality control surrounding data collection, and susceptibility to multiple sources of bias for comparing outcomes [12]—real-world data may provide guidance in understanding and exploiting the specific clinical properties of these two drug classes. Indeed, several real-world studies (RWS) have been recently carried out in different countries and populations mostly focusing on CV rather than kidney outcomes [13–19]; these studies have suggested an overall similar benefit of GLP-1 RA and SGLT-2i on CV composite endpoints [20].

In this study, we have analyzed the effectiveness and safety profile of first time-users of GLP-1 RA in comparison with first time-users of SGLT-2i from 2015 to 2020 in a routine clinical setting in Lombardy, the most populated Italian region.

## Methods

This analysis used linkable administrative health databases of the Lombardy region, which include demographic data of all residents and information on hospital records and drug prescriptions reimbursed by National health System. Data are available for about 10 million inhabitants of Lombardy from 2000 to 2020. Access to data is allowed within the agreement between the Istituto di Ricerche Farmacologiche Mario Negri (IRFMN) and Regional Health Ministry of Lombardy.

Subjects with T2D aged 50 years and older were included if they had at least two prescriptions of a GLP-1RA or a SGLT-2i (ATC code A10*) from January 1, 2015, to December 31, 2019 (from 2015 onward, a relevant number of SGLT-2i prescriptions was observed in the Italian market), and no prior exposure to any medications belonging to the same drug classes in the previous five years before entering study cohorts.

Subjects were split into two study cohorts according to the first exposure (index date) to GLP-1RA or SGLT-2i, respectively. A propensity score matching (PSM) in a 1:1 ratio of GLP-1 RA with SGLT-2i users was applied to reduce confounding due to imbalance in study covariates, considering all the variables reported in Table 1.

**Table 1:** Baseline characteristics of matched population by treatments status from 2015 to 2019

Variables	Cohorts		
	GLP-1 RA (N = 20,762)	SGLT-2i (N = 20,762)	Standardized differences
Mean age (+ SD)	66.30 ± 8.60	66.36 ± 8.25	− 0.00
Gender (Female)	8318 (40.1)	8334 (40.1)	− 0.00
Comorbidities of interest, n (%) (in the previous 5 years)			
Cerebrovascular disease	795 (3.8)	785 (3.8)	0.00
Cardiovascular disease	2659 (12.8)	2660 (12.8)	− 0.00
Heart failure	791 (3.8)	821 (4.0)	0.00
Peripheral vascular disease	655 (3.1)	655 (3.1)	0.00
Lower limb complication	172 (0.8)	180 (0.9)	− 0.00
Renal disease	347 (1.7)	337 (1.6)	0.00
Neuropathy	246 (1.2)	198 (0.9)	0.02
Diabetic retinopathy	17 (0.1)	17 (0.1)	0.00
Chronic obstructive pulmonary disease	502 (2.4)	481 (2.3)	0.00
Cancer	1513 (7.3)	1397 (6.7)	0.02
Antihyperglycemic drugs, n (%) (in the previous 5 years)			
GLP-1 RA	0 (0.0)	1399 (6.7)	− 0.38
SGLT-2i	0 (0.0)	0 (0.0)	0.00
Insulin	5429 (26.2)	5414 (26.1)	0.00
Other AHAs	19.834 (95.5)	19.609 (94.4)	0.04
Metformin	18,344 (88.3)	18.323 (88.2)	0.00
Sulfonylureas	10.142 (48.8)	10.167 (49.0)	− 0.00
Glinides	2128 (10.2)	2081 (10.0)	0.00
Glitazones	4289 (20.7)	4276 (20.6)	0.00
Acarbose	1739 (8.4)	1707 (8.2)	0.00
DDP-4i	7391 (35.6)	7402 (35.6)	− 0.00
N. of antihyperglycemic drug classes, median [IQR]	2.0 (1–3)	2.0 (1–3)	0.16
Patients with no previous anti-hyperglycemic drug treatment, n (%)	928 (4.5)	1148 (5.5)	0.04
Other medications of interest, n (%)			
Antihypertensive drugs	16.626 (80.1)	16.550 (79.7)	0.00
ACE-I/ARBS	14.044 (67.6)	14.002 (67.4)	0.00
Lipid lowering drugs	13.548 (65.2)	13.529 (65.2)	0.00
Antiplatelet drugs	6757 (32.5)	6731 (32.4)	0.00
Oral anticoagulant drugs	1426 (6.9)	1429 (6.9)	0.00
DDCI Index, median [IQR]	4 (2.7)	4 (2.7)	0.00
Hospital admission, mean (± SD)	1.2 (± 1.7)	1.2 (± 1.7)	0.00
Duration of diabetes, n (%)			
0–4	5106 (24.6)	5131 (24.7)	0.00
5–9	5185 (25.0)	5142 (24.8)	
10–14	5265 (25.4)	5242 (25.3)	
15 +	5206 (25.1)	5245 (25.3)	
Median [q1-q3]	10 (5–15)	10 (5–15)	0.00

*GLP-1 RA* glucon-like peptide-1 receptor agonists, *SGLT-2i* sodium glucose cotransporter 2 inhibitors

*AHAs* antihyperglycemic agents, *DPP-4i* dipeptidyl peptidase-4 inhibitors, *ACE-I* angiotensin-converting enzyme inhibitors

*ARBs* angiotensin II receptor agonist blockers, *DCCI* Drug Derived Complexity Index, *SD* standard deviation, *IQR* interquartile range

The adequacy of PSM was assessed by standardized mean difference (SDM) of post-matching patients’ characteristics. Good balance is conventionally set at SMD < 0.10. Information on history of comorbidities (collected through hospitalization), previous exposure to the medications of interest, any hospital admissions, Drug Derived Complexity Index (DDCI) [21], and duration of diabetes were gathered as reported in Table 1. A sensitivity analysis was performed on subjects treated with insulin in the 12 months before entering study cohort (Additional file 1). Hospitalization and pharmacy prescriptions were collected according to the international classification of diseases, ninth revision, and Anatomic Therapeutic Chemical classification code, respectively (Additional file 2, Appendix).

All subjects were followed-up starting from the first prescription of GLP-1RA or SGLT-2i until the occurrence of one of the following outcomes: death from any cause, hospital admission (as primary diagnosis) for myocardial infarction (MI), stroke (both ischemic or hemorrhagic), heart failure (HF), and renal disease as primary diagnosis (patients with advanced renal disease). Clinical events were also considered as composite outcomes (MACE-3: all cause-death, non-fatal MI, non-fatal stroke; MACE-4: all cause-death, non-fatal MI, non-fatal stroke, unstable angina). Safety was evaluated by retrieving data regarding the occurrence of hospitalization, as primary diagnosis, for hypoglycemia, ketoacidosis, diabetic coma, amputations, acute renal failure, syncope, and fractures.

### Statistical analysis

Descriptive statistic was reported as mean (± SD), number (%), or median (IQR). Outcomes were calculated as number of events and percentages and, the Cox proportional hazards regression model was used to estimate hazard ratio (HRs) with 95% confidence interval. Outcomes were analyzed by intention-to-treat (ITT) and stratified also according to pre-existing CV diseases (hospitalization for cardio-cerebrovascular disease or peripheral vascular disease in the five years before entering study cohort). Outcomes were adjusted for variables with a SDM ≥ 0.10. A further analysis was performed in a subgroup of subjects who were exposed to insulin in the 12-month period before entering study cohort and were excluded from the main analysis after PSM. In addition, a per protocol (PP) analysis was performed in patients who did not discontinue GLP-1RA or SGLT-2i (discontinuation was defined as no prescription for 7 months after the last refill).

## Results

A total of 61,431‬ first users of GLP-1RA or SGLT-2i were identified between January 1, 2015, and December 31, 2019: 21,960‬ (35.7%) in the GLP-1RA cohort and 39,471‬ (64.2%) in the SGLT-2i cohort. After the PSM, 20,762 patients per cohort were included in the analysis. All variables considered in the PSM were well-balanced (all standardized differences were < 0.1) except for higher previous exposure to GLP-1RA in SGLT-2i first-users (Table 1).

**Fig. 1:**
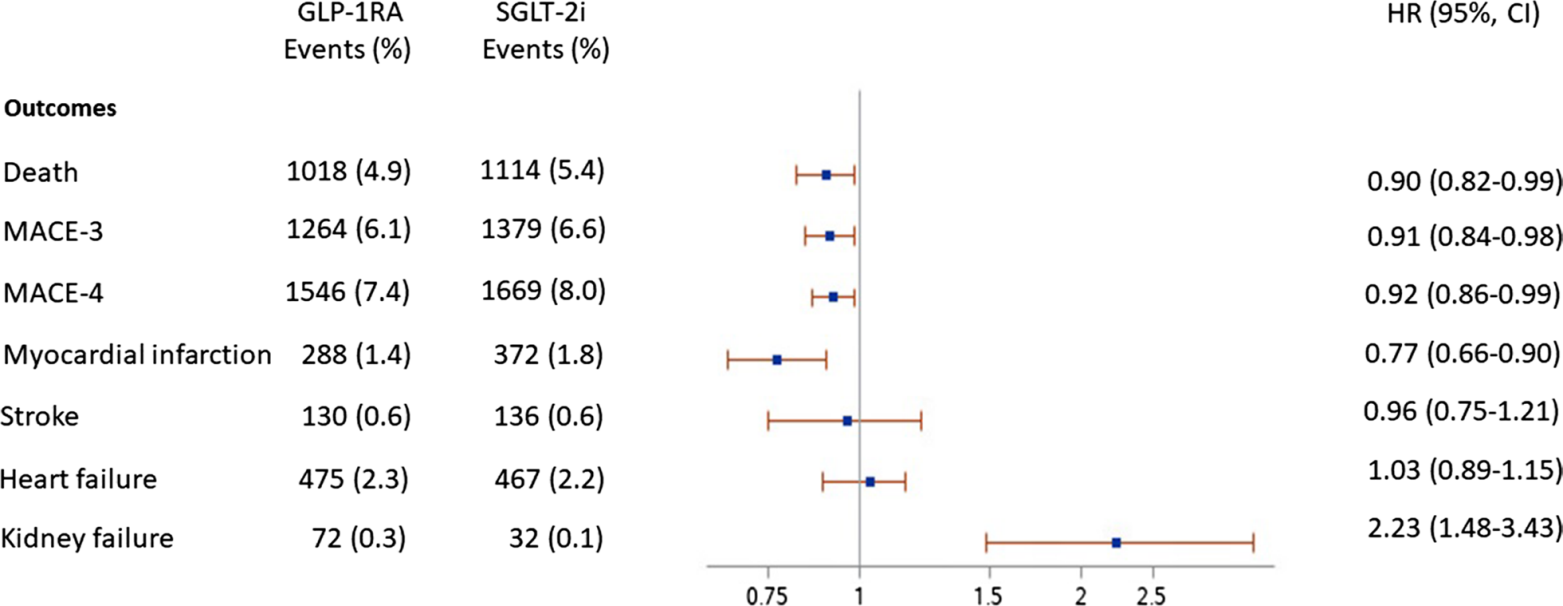
Number of events and hazard ratio (CI 95%), for death and clinical outcomes in matched populations according treatment status (ITT analysis). *GLP-1RA* Glucagon-like peptide-1 receptor agonists, *SGLT-2i* Sodium glucose transporter-2 inhibitors, *MACE-3* All cause death, non-fatal myocardial infarction, non-fatal stroke, *MACE-4* All-cause death, non-fatal myocardial infarction, non-fatal stroke, unstable angina

**Fig. 2:**
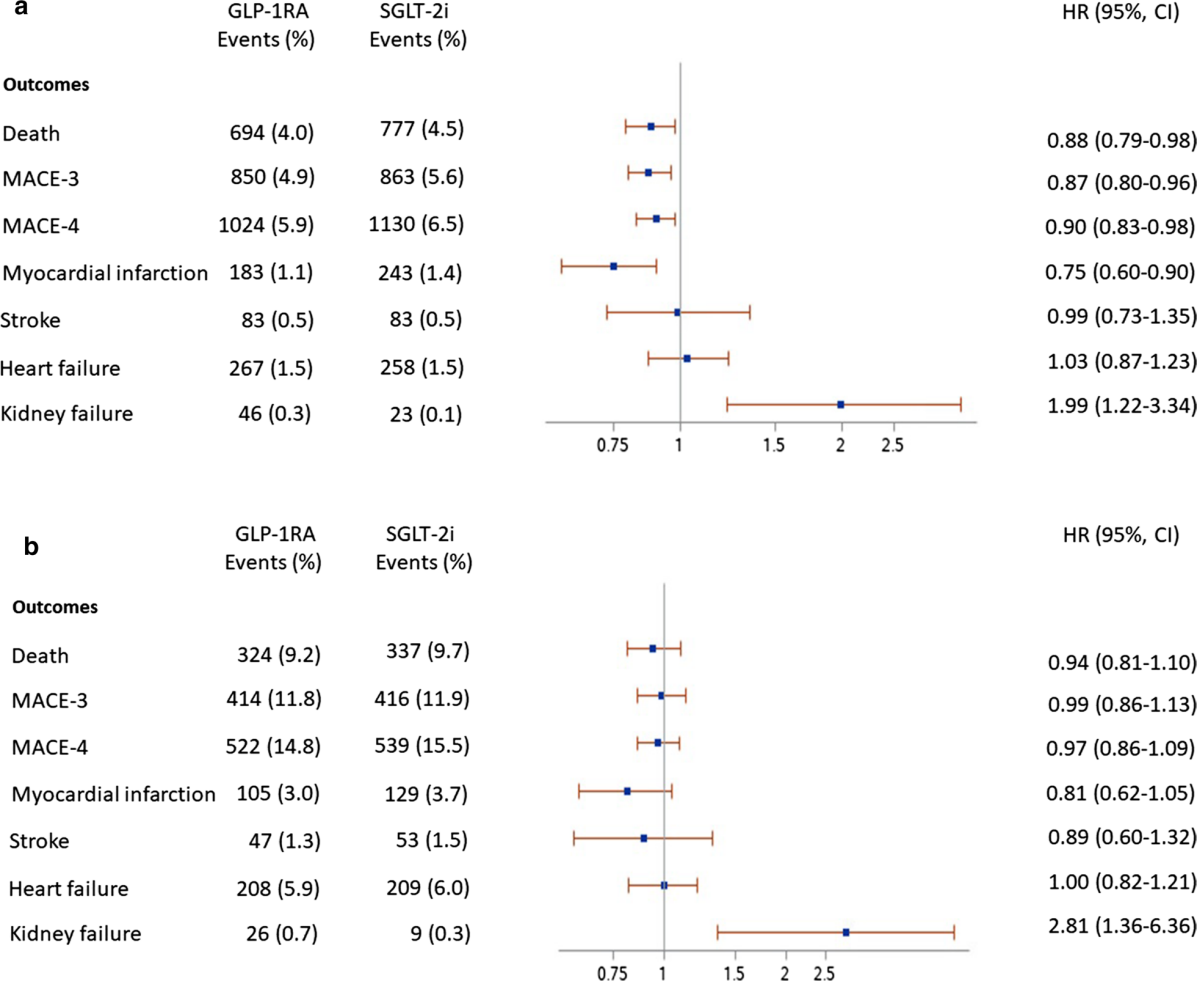
**A** Number of events and hazard ratio (CI 95%), for death and clinical outcomes in matched populations without previous cardiovascular disease according to treatment status**. B** Number of events and hazard ratio (CI 95%), for death and clinical outcomes in matched populations with previous cardiovascular disease according to treatment status. *GLP-1RA* glucagon-like peptide-1 receptor agonists, *SGLT-2i* sodium glucose transporter-2 inhibitors, *MACE-3* all cause death, non-fatal myocardial infarction, non-fatal stroke, *MACE-4* all-cause death, non-fatal myocardial infarction, non-fatal stroke, unstable angina

The mean follow-up time was similar for both cohorts (2.78 ± 1.30 years for the GLP-1RA cohort and 2.77 ± 1.37 years for the SGLT-2i cohort). The ITT analysis showed a significant risk reduction for non-fatal MI (HR 0.77; CI 95% 0.66–0.90), MACE-3 (HR 0.91; CI 95% 0.84–0.98), and MACE-4 (HR 0.92; CI 95% 0.86–0.99) in GLP-1RA vs. SGLT-2i first-users (Fig. 1). No difference was observed in the risk of hospitalization for HF and stroke between the two cohorts. A lower prevalence of being hospitalized for renal disease was observed in SGLT-2i first-users as compared to GLP-1RA first-users (0.1% vs. 0.3%). Notably, the risk reduction in death, MACE-3, MACE-4, and MI was evident in the subgroup of subjects without previous CV diseases as opposed to those with a previous hospitalization for CV diseases (Fig. 2A, B).

We also carried out a PP analysis in 58.1% GLP-1RA and 55.2% SGLT-2i first-users who remained adherent to treatment (Table 2). A similar trend in the risk reduction of MACE-4, as in the ITT analysis, was observed, and a statistically significant risk reduction was seen for MI (HR 0.70, 95% CI 0.57–0.85) and MACE-3 (HR 0.88, 95% CI 0.79–0.98) in the GLP-1RA cohort compared to the SGLT-2i cohort. As in the ITT analysis, GLP-1RA initiators were at higher risk of hospitalization for renal disease compared to SGLT-2i (HR 2.23, CI 95% 1.48–3.43).

**Table 2:** Number of events and hazard ratio (CI 95%), for death and clinical outcomes in matched populations according to treatment status (PP analysis)

Outcomes	Cohort		
	GLP-1 RA N = 12,062 N (%)	SGLT-2i N = 11,470 N (%)	HR (CI95%)
Death	528 (4.4)	555 (4.8)	0.89 (0.79–1.00)
Mace3	690 (5.7)	736 (6.4)	0.88 (0.79–0.98)
Mace4	881 (7.3)	917 (8.0)	0.91 (0.83–1.00)
Myocardial infarction	170 (1.4)	228 (2.0)	0.70 (0.57–0.85)
Stroke	71 (0.6)	82 (0.7)	0.82 (0.60–1.13)
Heart failure	277 (1.7)	238 (2.0)	1.11 (0.93–1.32)
Renal disease	36 (0.3)	11 (0.1)	3.04 (1.60–6.20)

*GLP-1RA* glucagon-like peptide-1 receptor agonists, *SGLT-2i* sodium glucose transporter-2 inhibitors;

*MACE-3* all cause death, non-fatal myocardial infarction, non-fatal stroke, *MACE-4* all-cause death, non-fatal myocardial infarction, non-fatal stroke, unstable angina

A subgroup of subjects treated with insulin in the 12-month period before entering study cohort, likely reflecting individuals with an advanced disease, was also computed after PSM (N = 5,529 in both cohorts; Additional file 1: Table S1). In this subgroup, after adjustment for the variables whose standardized differences were > 10%, including age, duration of diabetes, renal disease, and DDCI-Index, point estimates for the risk of MACE-3, MACE-4, MI and stroke favored new users of GLP-1RA versus SGLT-2i although this difference was not statistically significant (Additional file 1: Table S2).

During follow-up, the incidence of serious adverse events was relatively low in each group (i.e., less than 1%). Fractures (GLP-1 RA 1.16%; SGLT-2i 1.17%) and syncope (GLP-1 RA 0.34%; SGLT-2i 0.35%) were more frequently registered in both cohorts (Table 3).

**Table 3:** Frequency of serious adverse events according treatment status from 2015 to 2019

Events	Cohorts	
	GLP-1RA (N = 20,762) N (%)	SGLT-2i (N = 20,762) N (%)
Hypoglycemia	11 (0.05)	1 (0.00)
Ketoacidosis	14 (0.07)	18 (0.09)
Diabetic coma	5 (0.02)	7 (0.03)
Acute kidney failure	12 (0.06)	2 (0.01)
Syncope	70 (0.34)	72 (0.35)
Fractures	241 (1.16)	243 (1.17)
Amputations	53 (0.26)	67 (0.32)

*GLP-1RA* glucagon-like peptide-1 receptor agonists, *SGLT-2i* sodium glucose transporter-2 inhibitors;

*MACE-3* all cause death, non-fatal myocardial infarction, non-fatal stroke, *MACE-4* all-cause death, non-fatal myocardial infarction, non-fatal stroke, unstable angina

## Discussion

In this analysis, new users of GLP-1RA displayed a significantly lower risk of non-fatal MI, MACE-3, and MACE-4 with respect to SGLT-2i initiators. This benefit appeared to be driven by the favorable effect of GLP-1RA versus SGLT-2i in patients without established CV disease. The overall neutral effect of SGLT-2i on MACE reduction in patients in primary prevention had already been suggested by a meta-analysis of SGLT-2i CVOT [1]. On the other hand, while GLP-1RA failed to demonstrate significant CV protection in patients without overt CV disease in another comprehensive systematic review and meta-analysis [2], the REWIND trial, enrolling 68.5% of individuals without established CV disease, proved the CV superiority of dulaglutide compared to placebo in both primary and secondary prevention cohorts with a similar MACE HR (p for interaction = 0.97) [22]. Therefore, GLP-1RA may be more effective than SGLT-2i in reducing MACE over a large spectrum of CV risk.

It should be acknowledged that in the present study the definition of established CV disease differed from that used in CVOT, as only patients with a hospitalization for a CV event within the last 5 years were considered, likely resulting in an apparent increased CV risk of the cohort without overt CV disease. The CV protective effects of GLP-1RA have been extensively investigated in both pre-clinical studies as well as in human RCT and RWS [23], and their ability to ameliorate early phases of the atherosclerotic process, such as inflammation, endothelial dysfunction and intima-media thickening, as well as to improve circulating stem cells functions, has been suggested [24].

The anti-atherosclerotic effect might at least partially explain why GLP-1RA could reduce CV outcomes vs. SGLT-2i in people without overt CV disease. A study recently published by Wright et al. [25] showed that in comparison with other combination regimens, the use of GLP-1RA was associated with a 7% risk reduction of major adverse cardiac and cerebrovascular events in people with T2D without CV disease. However, an increasing body of evidence, coming mostly from preclinical studies, highlighted that also SGLT-2i might exert beneficial effects on inflammation, macrophage polarization and arterial-flow mediated dilation [26].

In a recent systematic review and meta-analysis of RWS, no differences in the occurrence of composite CV outcomes and MACE were found between new users of SGLT-2i and GLP-1RA [20]. Indeed, the studies by Patorno et al. [27], enrolling the largest population to date, and Longato et al. [13] were the only ones to detect a significantly lower risk of composite CV outcome occurrence (hospitalization for MI or stroke and all-cause death or myocardial infarction or stroke, respectively) in SGLT-2i compared to GLP-1RA new users. However, Patorno et al. and Longato et al. found a significant CV benefit in SGLT-2i initiators in comparison with GLP-1 RA initiators in the cohort of patients in secondary prevention. It should be noted that RWS, such as the retrospective observational study of Longato et al., differ in the definition of the investigated composite CV outcomes as well as the criteria according to which patients are defined as affected from established CV disease, making direct study comparisons difficult. For instance, in the study by Patorno et al., patients were considered as in secondary prevention if diagnosed with chronic ischemic heart disease or peripheral artery disease or related procedures, heart failure, ischemic stroke, or lower extremity amputation in the 12 months before inclusion [27].

In our analysis, in the subgroup of new users of GLP-1RA and SGLT-2i treated with insulin in the previous 12 months, likely at a more advanced stage of diabetes, both cohorts displayed a similar risk of CV events occurrence. In a meta-analysis of CVOT with SGLT-2i and GLP-1RA, patients on background insulin therapy had a higher CV event rate than those not on insulin [28], reinforcing the notion that GLP-1RA may be more effective than SGLT-2i only in patients without overt CV disease.

No significant difference in hospitalization for HF was found between the two cohorts, whereas in previous in RWS SGLT-2i seem to be associated with lower risk of heart failure and total mortality comparing new users of SGLT-2i and GLP-1RA [17–19]. SGLT-2i displayed an unparalleled benefit on HF-related outcomes in randomized controlled trials regardless of the baseline cardio-renal risk of the enrolled population [29]; accordingly, a meta-analysis of RWS showed that SGLT-2i were more effective than GLP-1RA on prevention of HF worsening [30]. On the other hand, albeit to a lesser extent, GLP-1RA have also been proven beneficial, significantly reducing HF hospitalization by 11% compared to placebo in CVOT [2] and by 12% compared to other glucose-lowering drugs except for SGLT-2i in RWS [20]. Interestingly, in three nested case–control studies involving 336,334 T2D patients without previous CV disease and HF, Wright et al. have recently shown that GLP-1RA were associated with a 18% risk reduction of HF compared to other glucose-lowering drugs [25]. However, Natali et al. recently reviewed the available clinical studies on GLP-1R agonism and left ventricular function, concluding that its role is still unclear in patients with both preserved or reduced left ventricle ejection fraction [30], despite the encouraging results of preclinical studies [31]. Further evidence is warranted to better understand the potential benefit of GLP-1RA in patients with HF; relevant to this question, the STEP-HFpEF trial is currently assessing the effect of semaglutide 2.4 mg once weekly in patients affected by HF with preserved ejection fraction (NCT04788511).

In our study, GLP-1RA initiators had a twofold risk to be hospitalized for renal disease compared to SGLT-2i. This result could be due to a higher prevalence of patients with a reduced renal function in GLP-1 RA group related to the different indications of these two drug classes. Indeed up to 2019 SGLT-2i could be prescribed only in patients with eGFR > 60 ml/min/1.73m^2^ while GLP-1 RA in those with eGFR up to 30 and more recently up to 15 ml/min/1.73m^2^. To date, only Lugner et al. investigated the renal composite outcome occurrence in GLP-1RA and SGLT-2i initiators, finding no significant differences between groups, even though point estimates for most kidney outcomes favoured SGLT-2i initiators [16]. Lugner et al. enrolled a population with preserved renal function (eGFR > 90 ml/min/1.73m^2^) [16], while the baseline eGFR of the patients included in the present study is unknown due to the use of claims data. Even though the proportion of patients with advanced and or acute renal disease at baseline was similar in the two cohorts according to the well-balanced PSM, we cannot exclude that SGLT-2i initiators could have had a better baseline renal function. The CVOT have been instructive on the SGLT-2i-mediated remarkable 38% reduction of hard kidney outcomes [1]*,* hence SGLT-2i are currently preferred in patients with CKD and albuminuria [32, 33]. Nonetheless, GLP-1RA were also able to reduce the risk of broad kidney endpoints including new-onset macroalbuminuria by 21% [2]; in addition, dulaglutide exhibited a significant reduction in the risk of eGFR decline in exploratory analyses of the REWIND and AWARD-7 trials [22, 34]. Specifically, in AWARD-7, dulaglutide was associated with a risk reduced the of 40% eGFR decline or end stage kidney disease. This was most evident among participants with macroalbuminuria, who had a 75% risk reduction, suggesting a potentially pronounced effect of dulaglutide to delay progression in participants with advanced CKD [34]. Finally, during follow-up, the incidence of serious adverse events (hypoglycemia, ketoacidosis, diabetic coma, amputations, acute kidney failure, syncope, and fractures) was relatively low in each group (less than 1%).

## Strengths and limitations

The large and unselected population, representing the real clinical setting, and the longer follow-up duration compared to similar studies [20] represent the main strengths of this study.

Also, besides the study specifically conducted in elderly patients by Patorno et al. [14], the population enrolled in this study was averagely older (mean age 66.3 years) than that of previous RWS (mean age ranging from 50 years in [15] to 63 years in [13], adding valuable insight on a subgroup of patients widely represented in everyday clinical practice.

A relevant limitation is represented by the possibility of residual confounding since clinical variables, such as HbA1c, eGFR, NYHA classification, body weight, and blood pressure, and the results of outpatient visits, were unavailable with claims analysis. Moreover, concomitant antihyperglycemic drugs may have changed during follow-up, potentially affecting patients’ CV and renal risk. In addition, analyzing data from a single Region cannot be generalized to the overall Italian population.

In conclusion, in the present study, GLP-1RA showed to be equally safe and more effective in reducing the risk of MACE-3, MACE-4 and MI when compared to SGLT-2i. On the other hand, GLP-1RA initiation was associated with a similar risk of hospitalization for HF and an increased risk of hospitalization for renal disease compared to SGLT-2i. This study adds to the growing body of real-world evidence, addressing the need to better understand the specific abilities of GLP-1RA and SGLT-2i in everyday clinical practice to tailor treatment to the individual patient.

## Supplementary Information

**Additional file 1: Table S1. **Baseline characteristics of matched population by treatment status from 2015 to 2019. **Table S2. **Events, incidence rate, hazard ratio (CI 95%) for death and clinical outcomes in matched populations previously treated with insulin, according to treatment status.

**Additional file 2:** Appendix.

## Data Availability

The data that support the findings of this study are available from Lombardy Region, but restrictions apply to the availability of these data, which were used under license for the current study, and so are not publicly available. Data are however available from the Lombardy Region upon reasonable request.
