# Supplementary material for: Effectiveness and safety of GLP-1 receptor agonists versus SGLT-2 inhibitors in type 2 diabetes: an Italian cohort study
Source: Cardiovasc Diabetol. 2022 Aug 24;21:162. doi: 10.1186/s12933-022-01572-y (PMC9400295; doi:10.1186/s12933-022-01572-y)
Supplement: Supplementary file 1 — Additional file 1: Table S1. Baseline characteristics of matched population by treatment status from 2015 to 2019. Table S2. Events, incidence rate, hazard ratio (CI 95%) for death and clinical outcomes in matched populations previously treated with insulin, according to treatment status. [file 12933_2022_1572_MOESM1_ESM.docx]

**Additional file 1**

**“****Effectiveness and Safety of GLP-1 Receptor Agonists versus SGLT-2 Inhibitors in Type 2 Diabetes: an Italian Cohort Study.**

Marta Baviera, Andreana Foresta, Pierluca Colacioppo, Maria Carla Roncaglioni, Mauro Tettamanti, Ida Fortino, Stefano Genovese, Irene Caruso, Francesco Giorgino

**Sensitivity Analysis:** Subjects treated with insulin in the 12 months before entering study cohort

**Table S1.** Baseline characteristics of matched population by treatment status from 2015 to 2019

|  | Cohorts | |  |
| --- | --- | --- | --- |
| Variables | GLP-1 RA  (N=5529) | SGLT-2i  (N=5529) | Standardized differences |
| Mean age (+SD) | 67.80 ± 9.08 | 66.05 ± 8.04 | 0.20 |
| Gender (Female) | 2395 (43.32) | 2147 (38.83) | 0.09 |
| Comorbidities of interest, n (%) |  |  |  |
| Cerebrovascular disease | 352 (6.37) | 264 (4.77) | 0.06 |
| Cardiovascular disease | 957 (17.31) | 1007 (18.21) | -0.02 |
| Heart failure | 442 (7.99) | 337 (6.10) | 0.07 |
| Peripheral vascular disease | 356 (6.44) | 340 (6.15) | 0.01 |
| Lower limb complication | 133 (2.41) | 121 (2.19) | 0.01 |
| Renal disease | 295 (5.34) | 93 (1.68) | 0.19 |
| Neuropathy | 142 (2.57) | 104 (1.88) | 0.04 |
| Diabetic retinopathy | 9 (0.16) | 15 (0.27) | -0.02 |
| Chronic obstructive pulmonary disease | 500 (9.04) | 403 (7.29) | 0.06 |
| Cancer | 1513 (7.29) | 1397 (6.73) | 0.06 |
| Antihyperglycemic drugs, n (%) |  |  |  |
| GLP-1RA | 0 (0.0) | 739 (13.37) | -0.55 |
| SGLT-2i | 0 (0.0) | 0 (0.0) | 0.00 |
| Other AHAs | 5529 (100.0) | 5529 (100.0) | 0.00 |
| Metformin | 4722 (85.40) | 4825 (87.27) | -0.05 |
| Sulfonylureas | 2907 (52.58) | 2721 (49.21) | 0.06 |
| Glinides | 881 (15.93) | 641 (11.59) | 0.12 |
| Glitazones | 1109 (20.06) | 1016 (18.38) | 0.04 |
| Acarbose | 662 (11.97) | 574 (10.38) | 0.05 |
| DDP-4i | 2183 (39.48) | 1489 (26.83) | 0.26 |
| No Antihyperglycemic drugs, n (%) | 0 (0.00) | 0 (0.00) | 0.00 |
| Medications of interest, n (%) |  |  |  |
| Antihypertensive drugs | 4640 (83.92) | 4585 (82.93) | 0.02 |
| ACE-I/ARBS | 3884 (70.25) | 3945 (71.35) | -0.02 |
| Lipid lowering drugs | 3861 (69.83) | 3878 (70.14) | -0.00 |
| Antiplatelet drugs | 2258 (40.84) | 2324 (42.03) | -0.02 |
| Oral anticoagulant drugs | 515 (9.31) | 398 (7.20) | 0.07 |
| DDCI Index, median [IQR] | 45 (3.8) | 45 (3.7) | 0.12 |
| Hospital admission, median [IQR] | 1 (0.2) | 1 (0.2) | 0.09 |
| Duration of diabetes, n (%) |  |  |  |
| 0-4 | 936 (16.93) | 670 (12.12) | 0.10 |
| 5-9 | 959 (17.34) | 864 (15.63) |  |
| 10-14 | 1338 (24.20) | 1447 (26.17) |  |
| 15+ | 2296 (41.53) | 2548 (46.08) |  |
| Median [q1-q3] | 13 (7,17) | 14 (9,16) | 0.15 |

**Table S2.** Events, incidence rate, hazard ratio (CI 95%) for death and clinical outcomes in matched populations previously treated with insulin, according to treatment status

|  | **Cohorts** | |  |
| --- | --- | --- | --- |
| Outcome  (Matched population) | **GLP-1 RA**  N (%) | **SGLT-2i**  N (%) | **HR (CI 95%)**  Adjusted***** |
| Death | 442 (8.1) | 455 (8.4) | 1.00 (0.87-1.14) |
| MACE-3 | 519 (9.6) | 563 (10.4) | 0.96 (0.85-1.08) |
| MACE-4 | 626 (11.5) | 686 (12.7) | 0.95 (0.85-1.06) |
| MI | 104 (1.9) | 146 (2.7) | 0.81 (0.63-1.04) |
| Stroke | 46 (0.8) | 63 (1.2) | 0.81 (0.55-1.19) |
| HF | 231 (4.2) | 212 (3.9) | 1.10 (0.91-1.33) |
| Renal disease | 35 (0.6) | 16 (0.3) | 2.37 (1.32-4.42) |

*Adjusted for the variables with standardized differences >10%: age, duration of diabetes, renal disease and DDCI.

Abbreviations: GLP-1RA: glucagon-like peptide-1 receptor agonists; SGLT-2i: sodium glucose transporter-2 inhibitors;

MACE-3: all cause death, non-fatal myocardial infarction, non-fatal stroke; MACE-4: all-cause death, non-fatal myocardial infarction, non-fatal stroke, unstable angina.
