# Supplementary material for: Effectiveness and safety of GLP-1 receptor agonists versus SGLT-2 inhibitors in type 2 diabetes: an Italian cohort study
Source: Cardiovasc Diabetol. 2022 Aug 24;21:162. doi: 10.1186/s12933-022-01572-y (PMC9400295; doi:10.1186/s12933-022-01572-y)
Supplement: Supplementary file 2 — Additional file 2: Appendix. [file 12933_2022_1572_MOESM2_ESM.docx]

**Additional file 2**

**“****Effectiveness and Safety of GLP-1 Receptor Agonists versus SGLT-2 Inhibitors in Type 2 Diabetes: an Italian Cohort Study.**

Marta Baviera, Andreana Foresta, Pierluca Colacioppo, Maria Carla Roncaglioni, Mauro Tettamanti, Ida Fortino, Stefano Genovese, Irene Caruso, Francesco Giorgino

**Appendix:**

**International Classification of Disease, Ninth Revision DM (ICD9-CM code) for diagnosis and procedure**

| **Disease** | **ICD-9-CM Code** |
| --- | --- |
| Cerebrovascular disease | 433.01; 433.11; 433.21; 433.31; 433.81; 433.91; 434.01; 434.91; 430; 431; 432; 432.0; 432.1; 432.9; 435; 435.0; 435.1; 435.2; 435.3; 435.8; 435.9; 436; 433.10; 433.20; 433.30; 433; 433.0; 433.00; 433.1; 433.10; 433.2; 433.20; 433.3; 433.30; 433.8; 433.80; 433.9; 433.90; 434; 434.0; 434.00; 434.9; 434.90; 437; 437.0; 437.1, 38.11, 38.12 |
| Cardiovascular disease | 414; 414.0; 414.00; 414.01; 414.02; 414.03; 414.04; 414.05; 414.1; 414.14; 414.19; 414.8; 414.9; 411; 411.0; 411.8; 411.81; 411.89; 410.x; 412; 413.x; 414.x; 36.x |
| Heart failure | 402.01; 402.11; 402.91; 404.01; 404.11; 404.91;  428, 428.0, 428.1; 428.9 |
| Peripheral artery disease | 440.2; 440.20; 440.21; 440.22; 440.23; 440.24; 440.29; 440.3; 440.30; 440.3; 440.32; 443.81; 250.7; 250.70; 250.71; 250.72; 250.73; 39.50; 38.18; 38.08; 39.90; 39.25; 39.26; 39.29; |
| Lower limb complication | 736 .70; 707.1; 440.23; 84.13; 84.15; 84.17; 84.11; 84.12; 730.06; 730.07; 730.16; 730.17; 730.26; 730.27; 730.86; 730.87; 730.96; 730.97; 681.1; 681.10; 681.9; 682.6; 682.7; 711.97; 785.4; 440.24 |
| Renal disease | 585.x; V451; 39.95; 54.98; V561; V562; V563; V563.1; V563.2; 38.95; 39.27; 39.42; 39.43; 581.81 |
| Neuropathy | 350; 350.0; 350.1; 350.2; 350.8; 350.9; 351; 351.0; 351.1; 351.8; 351.9; 354; 354.0; 354.1; 354.2; 354.3; 354.4; 354.5; 354.8; 354.9; 355; 355.0; 355.1; 355.2; 355.3; 355.4; 355.5; 355.6; 355.7; 355.71; 355.79; 355.8; 355.9; 378.51; 378.52; 378.53; 378.54; 357.2; 337.1 |
| Diabetic retinopathy | 362.0; 362.01; 362.02; 362.01 + 14.24; 362.02 + 14.24; 362.55; 361; 364.42; 365.63; 369.xx |
| Chronic obstructive pulmonary disease | 490, 491.x, 494.x, 496, 492.x |
| Cancer | Between 140 and 165, Between 170 and 208, Between 210 and 239, 2592 |
| Hypoglycemia | 2510; 2512 |
| Ketoacidosis | 2501; 25010; 25011; 25012; 25013 |
| Diabetic coma | 25030; 25031; 25032; 25033 |
| Acute kidney failure | 584; 5845; 5846; 5847; 5848; 5849 |
| Syncope | 9921; 7802 |
| Fractures | Between 800 and 829 |
| Amputation | 8411; 8412; 8413; 8415; 8417; 8411; 78411 |

[**Anatomical Therapeutic Chemical Classification**](https://en.wikipedia.org/wiki/Anatomical_Therapeutic_Chemical_Classification_System)

| **Drugs** | **ATC-code** |
| --- | --- |
| GLP-1RA | A10BX04; A10BX07; A10BX10; A10BX13;  A10BX14; A10BJ* |
| SGLT-2i | A10BK*; A10BX09; A10BX11; A10BX12;  A10BD15; A10BD16; A10BD19; A10BD20;  A10BD21 |
| Insulin | A10A*; A10AB*; A10AE*; A10AC*; A10AD* |
| Metformin | A10BA*; A10BD01; A10BD02; A10BD03; A10BD05; A10BD07; A10BD08; A10BD10; A10BD11; A10BD13; A10BD14; A10BD15; A10BD16; A10BD17; A10BD18; A10BD20 |
| Sulfonylureas | A10BB*; A10BD01; A10BD02; A10BD04;  A10BD06 |
| Glinides | A10BX02; A10BX03; A10BX08; A10BD14; |
| Glitazones | A10BG*; A10BD03; A10BD04; A10BD05;  A10BD06; A10BD09; A10BD12 |
| Acarbose | A10BF*; A10BD17 |
| DDP-4i | A10BH*; A10BD07; A10BD08; A10BD09;  A10BD10; A10BD11; A10BD12; A10BD13;  A10BD18; A10BD19; A10BD21; A10BH51 |
| Antihypertensive drugs | C02*; C03*; C03; C04*; C07*; C08*, C09*; C10BX03; C10BX09 |
| ACE-I/ARBs | C09*; C10BX10 |
| Lipid lowering drugs | C10* |
| Antiplatelets | N02BA01; B01AC*; C10BX08; C10BX02; C10BX05; C10BX01; |
| Oral anticoagulants | B01AA03; B01AA07;  B01AE07; B01AF01; B01AF02; B01AF03 |

GLP-1 RA: glucagon-like peptide-1 receptor agonists; SGLT-2i: sodium glucose transporter-2 inhibitors; DPP-4i: dipeptidyl peptidase 4 inhibitors; ACE-I: angiotensin-converting enzyme inhibitors; ARBs: angiotensin II receptor agonist blockers
